# A Short Dynamic Scan Method of Measuring Bone Metabolic Flux Using [^18^F]NaF PET

**DOI:** 10.3390/tomography7040053

**Published:** 2021-10-25

**Authors:** Tanuj Puri, Musib M. Siddique, Michelle L. Frost, Amelia E. B. Moore, Glen M. Blake

**Affiliations:** 1 Department of Biomedical Engineering, School of Biomedical Engineering and Imaging Sciences, King’s College London, 4th Floor Lambeth Wing, St. Thomas’ Hospital, London SE1 7EH, UK; tanujpuri82@gmail.com; 2 Radcliffe Department of Medicine, Level 6 West Wing, John Radcliffe Hospital, Headley Way, Headington, Oxford OX3 9DU, UK; musib_siddique@yahoo.co.uk; 3 Institute of Cancer Research Clinical Trials & Statistics Unit (ICR-CTSU), Institute of Cancer Research, 15 Cotswold Road, Sutton SM2 5NG, UK; michelle.frost@icr.ac.uk; 4 Department of Cancer Imaging, School of Biomedical Engineering and Imaging Sciences, King’s College London, 4th Floor Lambeth Wing, St. Thomas’ Hospital, London SE1 7EH, UK; amelia.moore@kcl.ac.uk

**Keywords:** positron emission tomography, PET, computed tomography, CT, [^18^F] sodium fluoride, [^18^F]NaF, short dynamic scan, bone metabolism, kinetic modelling, arterial input function

## Abstract

[^18^F]NaF PET measurements of bone metabolic flux (K_i_) are conventionally obtained with 60-min dynamic scans analysed using the Hawkins model. However, long scan times make this method expensive and uncomfortable for subjects. Therefore, we evaluated and compared measurements of K_i_ with shorter scan times analysed with fixed values of the Hawkins model rate constants. The scans were acquired in a trial in 30 postmenopausal women, half treated with teriparatide (TPT) and half untreated. Sixty-minute PET-CT scans of both hips were acquired at baseline and week 12 after injection with 180 MBq [^18^F]NaF. Scans were analysed using the Hawkins model by fitting bone time–activity curves at seven volumes of interest (VOIs) with a semi-population arterial input function. The model was re-run with fixed rate-constants for dynamic scan times from 0–12 min increasing in 4-min steps up to 0–60 min. Using the Hawkins model with fixed rate-constants, K_i_ measurements with statistical power equivalent or superior to conventionally analysed 60-min dynamic scans were obtained with scan times as short as 12 min.

## 1. Introduction

Hawkins et al. were the first to measure bone metabolic flux (K_i_) using 60-min fluorine-18 sodium fluoride ([^18^F]NaF) dynamic positron emission tomography (PET) scans [1]. The Hawkins model represents [^18^F]NaF bone tracer kinetics by a two-tissue compartmental model and uses the 0–60 min bone time–activity curve (TAC) and the arterial plasma input function (AIF) to estimate the constants K_1_, k_2_, k_3_, k_4_ and F_bv_. K_1_ and k_2_ represent the forward and backward tracer exchange rates between blood and extracellular fluid (ECF), k_3_ and k_4_ represent the forward and backward tracer exchange rates between ECF and bone mineral, and F_bv_ is the fractional blood volume within the bone volume of interest (VOI). K_1_ (units: mL min^−1^ mL^−1^) is widely regarded as a measurement of bone blood flow [2]. The rate constants k_2_, k_3_ and k_4_ are in units: min^−1^. K_i_ (units: mL min^−1^ mL^−1^) is the [^18^F]NaF plasma clearance to bone mineral calculated using the equation:

K_i_ = K_1_ ∗ k_3_/(k_2_ + k_3_), (1)

Since K_i_ varies with osteoblastic activity in the bone VOI [3] the measurement is sometimes referred to as bone metabolic flux [4].

The 60-min scan time makes the Hawkins method uncomfortable for subjects as well as makes the investigation expensive and takes up valuable scanning time [5]. Therefore, methods have been described for estimating K_i_ from a single short (4-min) static scan acquired around 60 min after tracer injection [6]. One advantage of the static scan method is that by acquiring multiple scans at different bed positions, K_i_ measurements can be made at several sites (for example, at the spine and hip) with one injection of the tracer. However, because no dynamic data are acquired during the first few minutes after injection, the static scan method cannot be used to measure K_1_ as well as K_i_.

This study aimed to analyse 60-min dynamic scan data to examine the practicality of using fixed population average values of k_2_, k_3_, k_4_ and F_bv_ to shorten the dynamic scan and obtain K_i_ measurements that are still as accurate and precise as those obtained from conventionally analysed 60-min scans with variable rate constants. In particular, we discuss whether, by eliminating the statistical errors in k_2_, k_3_ and k_4_ when fitting the 60-min scan data to the Hawkins model, such short-scan measurements of K_i_ may have equal or greater statistical power for measuring response to treatment than conventionally analysed 60-min dynamic scans.

## 2. Materials and Methods

### 2.1. Study Population

This report presents a retrospective analysis of data from a previously published trial of the effect of treatment of postmenopausal women with teriparatide (Forteo, Eli Lilly, Indianapolis, IN, USA), a synthetic parathyroid hormone with bone anabolic properties, on bone metabolic flux at the hip [7]. Written informed consent was obtained from all participants and the study was approved by the St Thomas’ Hospital Research Ethics Committee (Reference number 09/H0802/117) and UK Administration of Radioactive Substances Advisory Committee. In brief, 30 treatment-naïve women with osteopenia (defined as a bone mineral density T-score at the spine or hip between −1 and −2.5) were randomised into two groups. One group received calcium and vitamin D (control group, *n* = 15) and the other group received 20 μg/day teriparatide (TPT) daily plus calcium and vitamin D for 12 weeks (TPT group, *n* = 15). Three subjects (2 from the TPT group and 1 from the control group) withdrew consent after randomisation and prior to treatment, so data from these subjects were excluded from the analysis.

### 2.2. PET Acquisition, Regions of Interest and Arterial Input Function

PET images of both hips were acquired using a GE Discovery PET-CT scanner (General Electric Medical Systems, Waukesha, WI, USA) with a 15.4 cm field of view (FOV). After a low-dose computed tomography (CT) scan, subjects were injected with 180 MBq [^18^F]NaF and a 60-min dynamic PET scan acquired consisting of twenty-four 5-s, four 30-s and fourteen 240 s frames. Venous blood samples were obtained at 30, 40, 50 and 60 min, and the tracer concentration in plasma was measured in a well-counter that was cross-calibrated with the PET scanner. All activity measurements were corrected for radioactive decay back to the time of injection. Attenuation correction was applied using the CT data and PET images reconstructed by filtered back-projection using a Hanning 6.3 mm filter. PET image matrix sizes in x, y, and z-direction were 128 × 128 × 47, and voxel sizes were 2.734 × 2.734 × 3.27 mm^3^. CT image matrix sizes were 512 × 512 × 47, and voxel sizes were 0.977 × 0.977 × 3.27 mm^3^.

Skeletal VOIs were first outlined on CT images and then transferred to PET to measure the bone TACs. Seven VOIs were defined [8]: (1) femoral shaft (FS): a cylindrical 60-mm section of bone in the upper femoral shaft; (2) intertrochanteric (ITR) with a 2D projection matching the dual-energy X-ray absorptiometry (DXA) ITR region of interest (ROI); (3) femoral neck (FN) matching the DXA FN ROI; (4) total hip (TH) matching the DXA TH ROI; (5) cortical bone (CORT): an annular cylindrical section similar to the FS but excluding the medullary cavity; (6) trabecular bone (TRAB): an ellipsoidal VOI within the ITR region including only trabecular bone; (7) pelvis (PELV): a VOI including the entire pelvis within the PET scan FOV. For the hip VOIs, the mean of the left and right sides was used for the analysis.

The plasma AIF was estimated using a semi-population method [9] derived from direct arterial sampling in ten postmenopausal women [10]. The venous blood samples obtained during the 60-min dynamic scan were used to define each subject’s terminal exponential. The mean population residual curve obtained from the ten women representing the fast exponential components was scaled for injected activity and, after adjusting the peak count rate to the time determined from a VOI drawn over the femoral artery, the scaled and time-adjusted population residual was added to the individual’s terminal exponential curve to obtain the 60-min AIF used for kinetic analysis [9].

### 2.3. Kinetic Modelling

**Figure 1:**
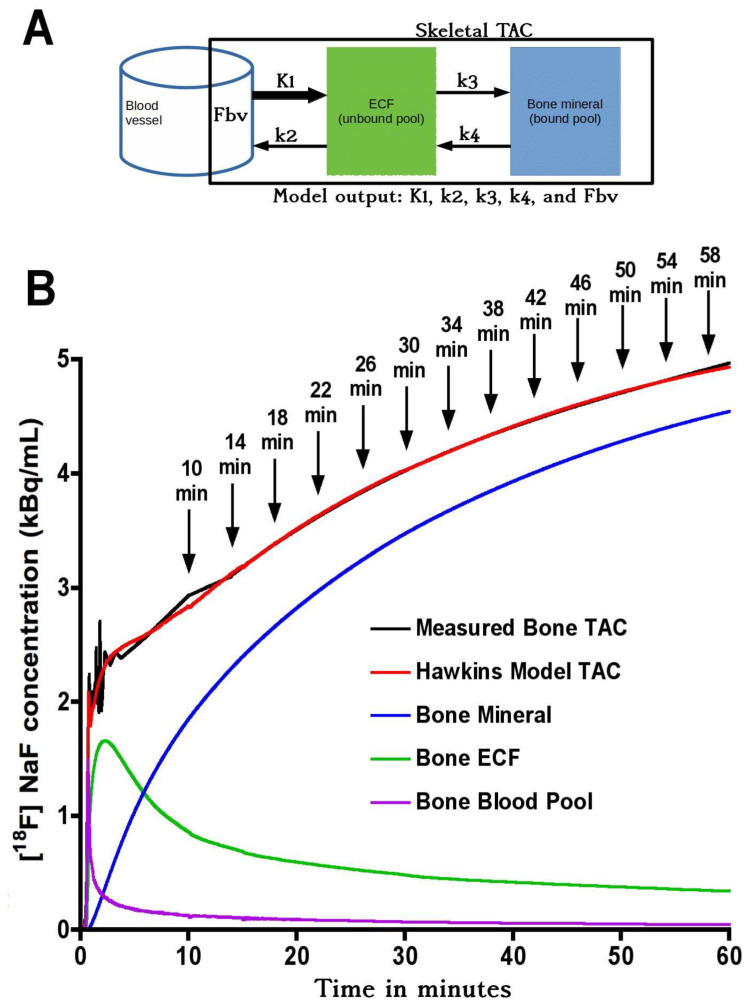
(**A**) Hawkins model with extracellular fluid (ECF) and bone mineral compartments. The black rectangular boundary shows the skeletal volume of interest (VOI) used to obtain the bone time–activity curve (TAC), which includes the fractional blood volume (F_bv_). K_1_ and k_2_ represent the forward and backward tracer exchange rates between blood and ECF, and k_3_ and k_4_ represent the forward and backward tracer exchange rates between ECF and bone mineral. Values of bone metabolic flux (K_i_) were calculated using Equation (1). (**B**) Variation of tracer concentration in different compartments of the Hawkins model between 0 and 60 min. Times marked with arrows show the mid-times of successive 4-min frames. Short-scan values of K_i_ were calculated for each 4-min frame with mid-times between 10 and 58 min (corresponding to full dynamic scan times between 12 and 60 min).

(a) Hawkins model 60-min dynamic scan K_i_: The bone TAC and AIF between 0–60 min were fitted to the Hawkins model using non-linear regression to obtain the five constants K_1_, k_2_, k_3_, k_4_ and F_bv_ (Figure 1). Values of K_1_, k_2_ and k_3_ were restricted between 0 and 1, and k_4_ between 0 and 0.05. The bone metabolic flux in each VOI was obtained from Equation (1). The quality of the curve fit to each bone TAC was evaluated using the Akaike information criterion (AIC) [11]. The K_i_ values obtained from the full 60-min dynamic scan data were considered the true values against which the outcomes of the short-scan evaluations were compared.

(b) Short-scan estimates of K_i_: In the short-scan analysis, shorter sections of the 60-min dynamic scan were re-analysed using fixed population average values of k_2_, k_3_, k_4_ and F_bv_ obtained from the full 60-min kinetic analysis described above. Population values were averaged across all seven VOIs and are listed in Table 1. The bone TAC and AIF were used to fit the Hawkins model equations in short sections from 0–12 min to 0–60 min incrementing one 4-min frame at a time (Figure 1B). The times in Figure 1B from 10 to 58 min refer to the mid-point of their respective 4-min frames. At each time point, K_i_ was defined as the value that gave the best least-squares fit to all the 4-min frames up to that time. At each trial value of K_i_, the value of K_1_ was set by inverting Equation (1) using the fixed values of k_2_ and k_3_. We note that the values of K_1_ assumed in this method are in a fixed ratio to K_i_ and do not represent true measurements of bone blood flow. However, valid measurements of K_1_ can be obtained by fitting the first two minutes of the dynamic scan data to a one-tissue compartmental model involving an exchange between the blood pool and ECF space. We have not performed this analysis in the present study, but note that for evaluating K_1_ an image derived AIF is preferable to the semi-population AIF used for the present K_i_ analysis [9].

### 2.4. Statistical Analysis

Ratios of short-scan K_i_ to the 60-min Hawkins model values for all 54 baseline and 12-week PET scans at all 7 VOIs (a total of 378 data points at each time point) were plotted against the short-scan duration in 4-min intervals from 12 to 60 min. To better understand the reasons for the differences between the two K_i_ values, at five representative times (12, 20, 32, 44 and 60 min) the same ratios were plotted against the values of k_3_ obtained from the 60-min Hawkins model analysis and non-linear regression curve fits obtained using a web-based programme [12]. Residuals from the k_3_ curve fit were then plotted against values of k_2_ and a second curve fit was obtained. Finally, residuals from the k_2_ curve fit were plotted against k_4_.

To assess statistical agreement between short-scan K_i_ and 60-min Hawkins model values in the clinical trial, scatter and Bland-Altman plots were obtained at each time point and Pearson correlation coefficients and 95% limits of agreement (LOA) were evaluated. To compare the statistical power of short-scan K_i_ and conventional 60-min Hawkins model values to evaluate response to TPT treatment, the means and standard errors of the percentage (%) changes between baseline and 12 weeks in the TPT and the control group were compared for each VOI at each short-scan time point. Values of Student’s t were used to assess the statistical significance of the response to TPT treatment at each short-scan time point for each VOI and compared with the equivalent Student’s t results for the conventional 60-min Hawkins model analyses.

To evaluate reasons for the differences in statistical power between the 60-min Hawkins model and short-scan analyses, scatter and Bland-Altman plots were drawn of the freely fitted values of k_2_, k_3_ and k_4_ obtained from Hawkins model analyses of the 60-min 12-week scans against the results obtained at baseline. Assuming that differences in the rate constants between baseline and 12-week scans were due to random measurement errors in the model parameters, the effects of these errors on follow-up 60-min Hawkins model K_i_ values were evaluated and compared with the differences between the 60-min Hawkins model and short-scan measurements of K_i_.

## 3. Results

**Figure 2:**
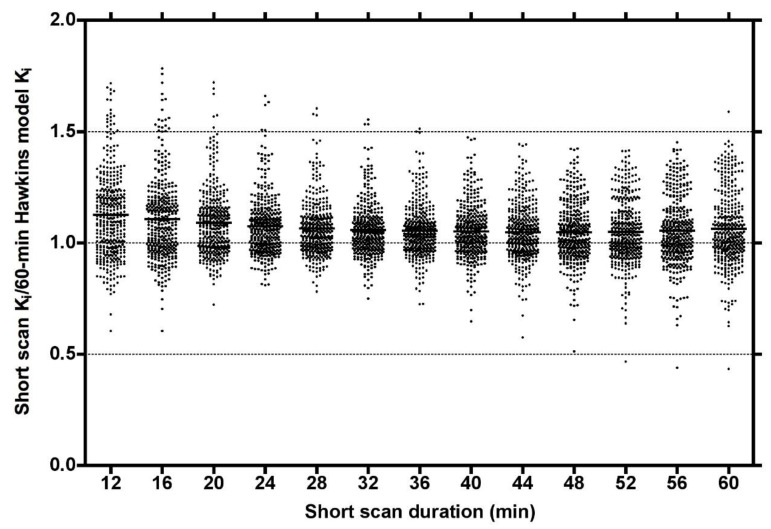
Plot of the ratios of short dynamic scan K_i_ values to the 60-min Hawkins model values for short-scan times between 12 and 60 min incremented in steps of 4 min. Data at each time point are for 54 [^18^F]NaF PET hip scans (27 baseline and 27 follow-up scans) with 7 volumes of interest analysed for each scan giving a total of 378 data points at each time point.

Plots of the ratio of short-scan K_i_ to the 60-min Hawkins model values for all scans at all VOIs for short dynamic scan times in increments from 12 to 60 min showed a range of values, which at their narrowest at around 32 min varied from 0.75 to 1.5 and had a small bias for short-scan K_i_ to overestimate the 60-min Hawkins model results (Figure 2).

**Figure 3:**
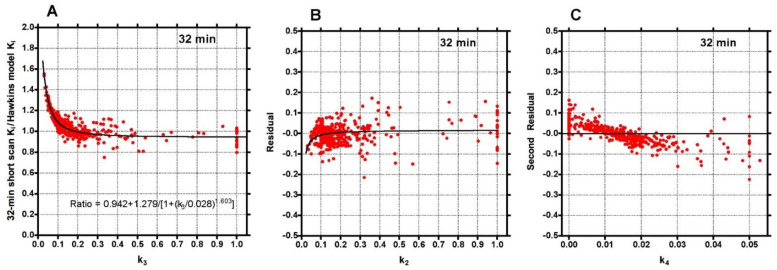
(**A**) Ratios of the 32-min short-scan K_i_ values to the 60-min Hawkins model values shown in Figure 2 plotted against the Hawkins model k_3_ values. The continuous black line is the least-squares fit to the 378 data points. (**B**) Residuals from the curve fit in (**A**) plotted against the Hawkins model k_2_ values. The continuous black line is the least-squares fit to the data points. (**C**) Residuals from the curve fit in (**B**) plotted against the Hawkins model k_4_ values.

To investigate the factors behind the dispersion of data points in Figure 2, the 32-min ratios were plotted against the conventionally analysed 60-min Hawkins model k_3_ values (Figure 3A). The fitted curve crossed unity at k_3_ = 0.18 min^−1^, equal to the fixed value of k_3_ assumed for the short-scan analysis (Table 1). For scans with conventional 60-min Hawkins model k_3_ values <0.18 min^−1^, ratios were systematically greater than unity, rising to 1.5 for the smallest k_3_ values, while for scans with k_3_ values >0.18 min^−1^ the ratios tended asymptotically to 0.94. When the residuals from the curve fit in Figure 3A were plotted against the 60-min Hawkins model k_2_ values a smaller inverted trend was found (Figure 3B). Finally, when the residuals from the k_2_ curve fit in Figure 3B were plotted against the 60-min Hawkins model k_4_ values there was a trend for points with k_4_ values less than the fixed k_4_ value of 0.014 min^−1^ to have positive residuals and points with k_4_ values >0.014 min^−1^ to have negative residuals (Figure 3C). Similar results were obtained when the short-scan ratios at 12, 20, 44 and 60 min were plotted against k_3_, k_2_ and k_4_ in the same way.

**Figure 4:**
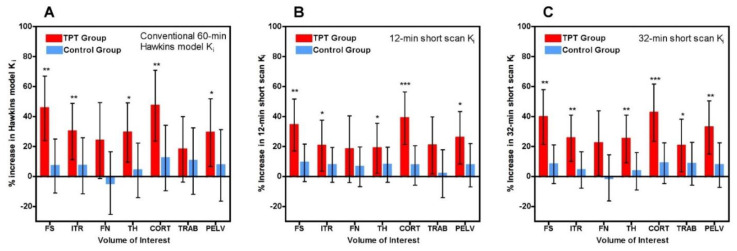
(**A**) Mean percentage change in 60-min Hawkins model K_i_ values between baseline and week 12 for teriparatide (TPT) treated and control groups for each of the seven volumes of interest (VOI) (FS: femoral shaft VOI; ITR: intertrochanteric VOI; FN: femoral neck VOI; TH: total hip VOI; CORT: cortical bone VOI; TRAB: trabecular bone VOI; PELV: pelvis VOI). Error bars are 95% CI. (**B**) Same as (**A**) but for the 12-min short-scan K_i_ data. (**C**) Same as (**B**) but for the 32-min short-scan K_i_ data. * *p* < 0.05 relative to baseline; ** *p* < 0.01; *** *p* < 0.001.

**Figure A1:**
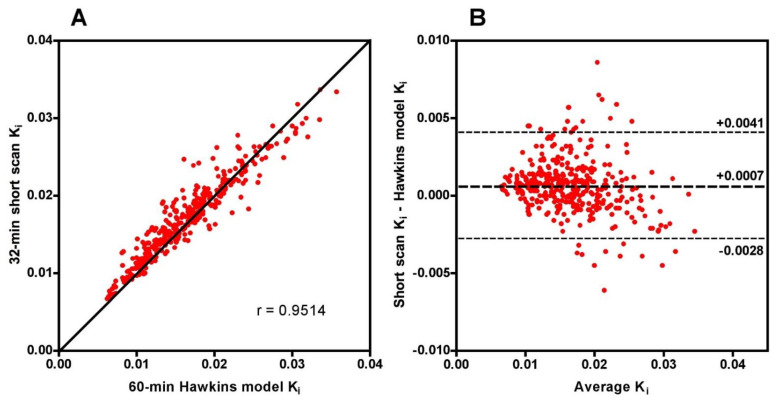
(**A**) Scatter and (**B**) Bland-Altman plots of the 32-min short-scan K_i_ and the 60-min Hawkins model values for the data plotted in Figure 3. The 378 data points in each plot were derived from 54 PET scans (27 baseline and 27 follow-up scans) with 7 volumes of interest analysed on each scan.

Scatter and Bland-Altman plots of short-scan K_i_ values against the conventional 60-min Hawkins model results showed dispersions consistent with the findings presented above with a correlation coefficient r = 0.951 and 95% LOA of −0.0028 to +0.0041 mL min^−1^ mL^−1^ at 32 min (Figure A1). The question arises whether the dispersion seen in this figure means that short-scan K_i_ results are less accurate measures of subjects’ response to treatment than conventional 60-min dynamic scan Hawkins model measurements of K_i_, or whether by fixing the values of k_2_, k_3_ and k_4_ a source of unwanted noise in the 60-min dynamic scan measurements is eliminated and the short-scan results are the more robust measurement. To examine this point, we compared the mean percentage changes in 60-min Hawkins model K_i_ values in the TPT and control groups in each of the seven VOIs (Figure 4A) with the corresponding results for the 12-min (Figure 4B) and 32-min (Figure 4C) short-scan K_i_ values. Figure 4 is annotated to show VOIs with *p* < 0.05, <0.01 and <0.001 compared with baseline. Comparison of Figure 4B,C with Figure 4A in terms of these *p*-value thresholds suggests that overall the 12-min short-scan K_i_ analysis performed as well as the 60-min Hawkins model analysis while the 32-min analysis performed better.

**Figure 5:**
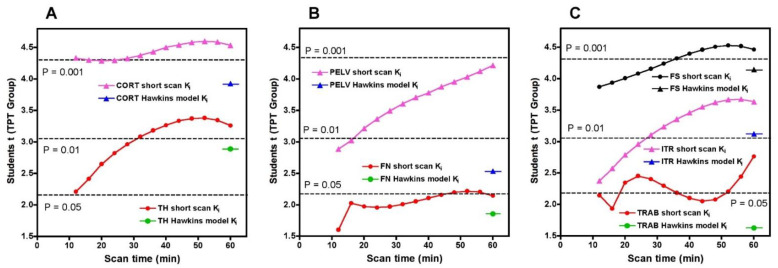
Values of Student’s t for the changes in the short-scan measurements of Ki between baseline and 12-weeks in the TPT group for scan times between 12 and 60 min. Results are shown separately for each of the seven volumes of interest (VOI). (**A**) CORT: cortical bone VOI; TH: total hip VOI; (**B**) FN: femoral neck VOI; PELV: pelvis VOI; (**C**) FS: femoral shaft VOI; ITR: intertrochanteric VOI; TRAB: trabecular bone VOI. For each VOI, the value of Student’s t for the 60-min Hawkins model analysis is shown for comparison. Dotted lines indicate values of Student’s t for *p* = 0.05, *p* = 0.01 and *p* = 0.001.

To compare the statistical significance of the short-scan and 60-min Hawkins results over the full range of short-scan times, we calculated Student’s t for the changes between baseline and 12-weeks in the TPT arm at each time point between 12 and 60 min and compared it against Student’s t for the conventionally analysed 60-min Hawkins data at each of the seven VOIs (Figure 5). For the CORT, PELV and TRAB VOIs, short-scan K_i_ results had higher Student’s t values at all scan times from 12 to 60 min. For the FN VOI, short-scan K_i_ had higher Student’s t at all times except the 12-min scan. For the remaining three VOIs (TH, FS and ITR), short-scan K_i_ had higher Student’s t at all scan times from 32 to 60 min.

**Figure A2:**
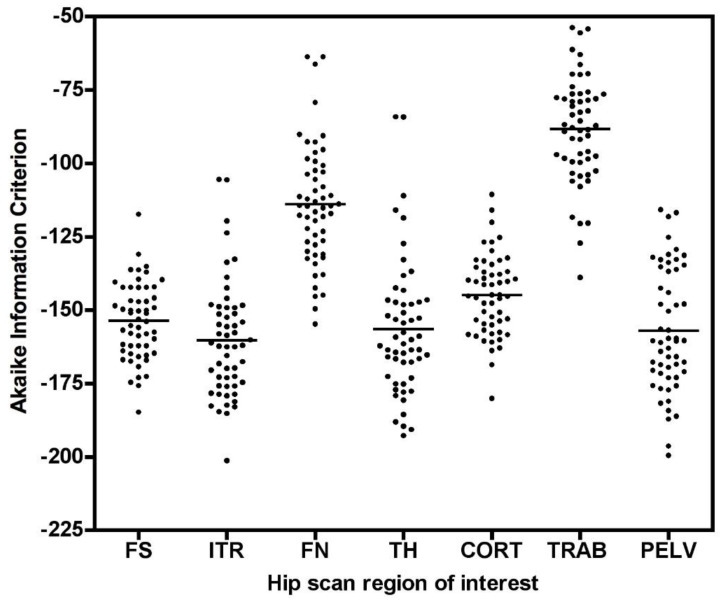
Akaike information criterion (AIC) results for the 0–60min PET scan Hawkins model curve fits for all volumes of interest (VOI) (FS: femoral shaft VOI; ITR: intertrochanteric VOI; FN: femoral neck VOI; TH: total hip VOI; CORT: cortical bone VOI; TRAB: trabecular bone VOI; PELV: pelvis VOI). Data at each VOI are for both the baseline and 12-week follow-up scans.

As shown in Figure 5, Student’s t results for the FN and TRAB VOIs were smaller than at other sites. This is due to a recognised technical problem with hip [18F]NaF PET scans where image streaking can occur in VOIs close to high activity in the urinary bladder, distorting the bone TAC [13]. The presence of this effect is demonstrated by the AIC data for the Hawkins model fits to the 60-min scan data (Figure A2), with AIC results showing systematically poorer curve fits for the FN and TRAB VOIs compared with the other sites.

**Figure 6:**
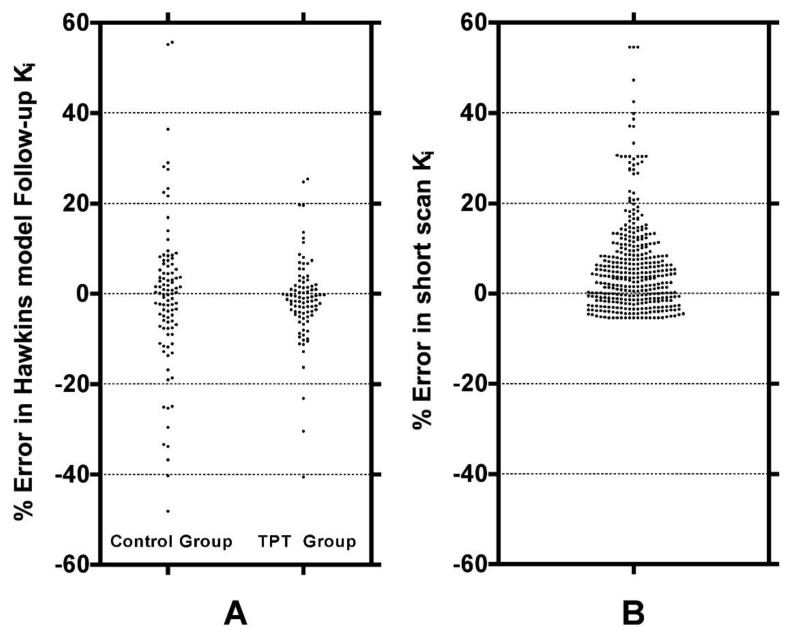
(**A**) The percentage changes in the Hawkins model 60-min dynamic scan K_i_ measurements between baseline and 12-week follow-up scans explained by the differences in the Hawkins model k_3_ parameter between the two scans. The distribution is shown separately for the control and TPT groups. The predicted changes were calculated from the curve fit in Figure 3A. (**B**) Percentage differences between the 32-min short-scan K_i_ measurements and the Hawkins model 60-min dynamic scan K_i_ values assuming that the freely fitted values of k_3_ are correct.

**Figure A3:**
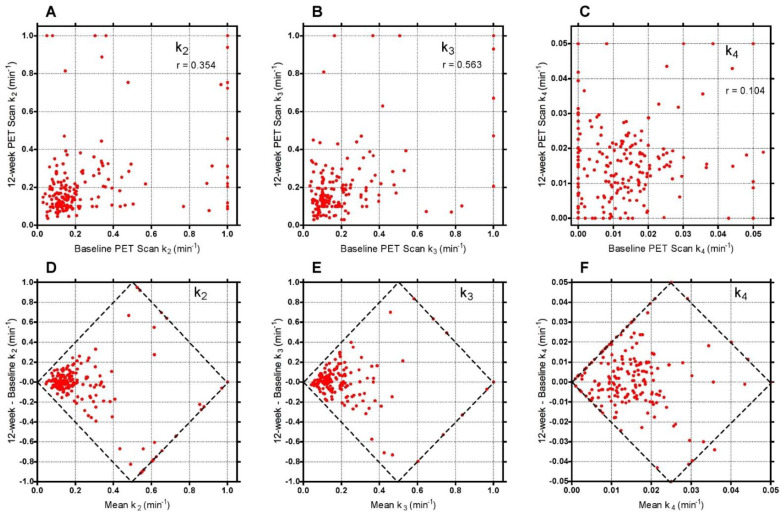
Scatter plots of 60-min Hawkins model k-number values for the baseline and 12-week scans for: (**A**) k_2_; (**B**) k_3_; (**C**) k_4_. The graphs in (**D**–**F**) show the respective Bland-Altman plots. The diamond-shaped dashed lines show the limits imposed by the restrictions placed on the values of the three rate constants. Points in this figure with k_2_ or k_3_ > 0.5 min^−1^ or k_4_ > 0.03 min^−1^ are predominantly measurements at the FN or TRAB VOIs with poor AIC.

To evaluate reasons for the good statistical performance of the short-scan data compared with the findings of conventionally analysed 60-min dynamic scans in Figure 5, scatter and Bland-Altman plots were drawn of the freely fitted Hawkins model k_2_, k_3_ and k_4_ results measured from the 12-week 60-min scans against the equivalent baseline measurements (Figure A3). Points in this figure with k_2_ or k_3_ > 0.5 min^−1^ or k_4_ > 0.03 min^−1^ are predominantly measurements at the FN or TRAB VOIs with poor AIC. Assuming that the differences in the rate constants between baseline and follow-up scans are due to random measurement errors, we evaluated the effect of the differences in k_3_ on the percentage changes in the baseline to the 12-week ratio in the 60-min Hawkins model K_i_ using the curve fit in Figure 3A. Figure 6A shows these results plotted separately for the control and TPT arms. The two distributions can be compared with the ratios of 32-min short-scan K_i_ to conventional 60-min Hawkins model K_i_ plotted in Figure 6B.

## 4. Discussion

PET imaging with [^18^F]NaF can play an important role during the early development of new treatments for osteoporosis by measuring changes in site specific bone formation rate [3] as early as three months after commencement of treatment, thereby aiding pharmaceutical companies to decide whether new drugs should proceed to further trials [7]. As the fracture site associated with the highest mortality and morbidity, the hip is an important target for these studies. However, the long acquisition time makes the conventional 60-min scan uncomfortable for clinical trial participants, is an expensive item in the research budget and competes for scanning time with routine clinical imaging services in high demand. In this study, we investigated an alternative approach using different dynamic scan times between 12 and 60 min analysed using the Hawkins model with fixed rate-constants and obtained K_i_ measurements with as good or better statistical power for detecting response to treatment as conventionally analysed 60-min scans with freely fitted rate constants. These results demonstrate the potential advantage of using fixed rate-constants for Hawkins model analyses.

Although short-scan K_i_ values in Figure 2 appear to systematically overestimate conventional 60-min Hawkins model values with a mean bias of approximately 5% at scan times around 30 min, this bias is explained by the non-linear curve fitted to the data in Figure 3A. In this figure, the short-scan and 60-min Hawkins model K_i_ values become equal with no bias at the short-scan fixed k_3_ value of 0.187 min^−1^. The fitted curve equals 1.05 at k_3_ = 0.124 min^−1^, and choosing this latter as the fixed value of k_3_ would remove the 5% bias noted above. The trends seen in Figure 3 are explained by the effects of changes in the Hawkins model rate constants k_2_, k_3_ and k_4_ on the TACs for the bone mineral compartment (the blue curve in Figure 1B) and the bone ECF compartment (the green curve in Figure 1B). As k_3_ increases, at later time points in Figure 1B, a greater proportion of the [^18^F]NaF tracer in the bone VOI resides in the bone mineral compartment and less in the bone ECF compartment. Hence, the value of the 60-min Hawkins model K_i_ increases at higher k_3_ values and, relative to the short-scan K_i_ value calculated with a fixed value of k_3_, the ratio plotted on the vertical axis in Figure 3A decreases. A detailed explanation of the trends shown in Figure 3 is complicated by the correlations between the three rate constants obtained from the curve fits to the 60-min bone VOI TACs. Both k_2_ (r = 0.557, *p* < 0.001) and k_4_ (r = 0.403, *p* < 0.001) correlate positively with k_3_. Additionally, k_3_ (r = 0.227, *p* < 0.001) and k_4_ (r = 0.490, *p* < 0.001) both correlate with K_i_. Given these relationships, it seems likely that the strong trend seen in Figure 3A already incorporates some of the dependence of K_i_ on k_2_ and k_4_ as well as k_3_ and may explain the weaker trends seen in Figure 3B,C.

A technical limitation of dynamic [^18^F]NaF PET imaging of the hip is the time-varying streaking artefacts sometimes seen in VOIs in close proximity to the bladder [13]. In the present study, this effect is seen in the systematically poorer AIC values to the Hawkins model fits for the FN and TRAB sites compared with the other five sites. This explains most of the outlier points with high k-numbers seen in Figure 3 and some of the outliers in Figure 2. It also explains why these VOIs performed less well at measuring the treatment effect in Figure 4 and Figure 5.

The difference between short-scan values of K_i_ calculated with fixed values of the Hawkins model rate constants and conventional 60-min dynamic scan values with freely fitted values raises the question of which method is optimal for clinical trial studies. This is a pragmatic question: the better method for clinical trials is the one with the best statistical power to discriminate changes in K_i_ values due to therapies that modify bone metabolism. The optimum method can be identified as the one that gives *p*-values with higher statistical significance or allows research studies to achieve the same *p*-value with fewer participants. Figure 4 demonstrates that short-scan results with scan times around 30 min gave similar % changes in K_i_ values as conventionally analysed 60-min scans but with smaller *p*-values at four of the seven sites. Figure 5 shows that as assessed by a Student’s t analysis, all seven sites performed better at 60-min if analysed using fixed values of the Hawkins model rate constants rather than freely fitted values. At three sites a 12-min short-scan performed better than the conventional 60-min analysis. The above findings suggest that the poor repeatability of the Hawkins model rate constants apparent in Figure A3 is a contributory factor to imprecision in the resulting K_i_ values and that any errors entailed in the short-scan method using fixed rate-constants may be preferable to the random measurement errors in the freely fitted 60-min Hawkins rate constants. Figure 6 demonstrates that scan-to-scan changes in 60-min Hawkins model K_i_ results explained by the relationship with k_3_ shown in Figure 3A are comparable to the differences between the short-scan K_i_ values calculated with fixed k-numbers and the conventional Hawkins model analysis with freely fitted k-numbers.

Previous authors have examined the advantages of short dynamic PET scans. Torizuka et al. [14] proposed a short dynamic [^18^F]FDG PET scan and found a good correlation between the K_i_ values obtained from 30-min and 60-min scans in patients with lung cancer. However, their study did not examine the effect of the shorter scan time on the statistical analysis of longitudinal studies. Strauss et al. [15] compared the use of a 10-min dynamic [^18^F]FDG PET scan combined with a static scan at 60 min for predicting individual k-values (K_1_, k_2_, k_3_, k_4_, F_bv_, K_i_) in tumours compared with a full 60-min dynamic scan. Again, although good correlations were reported, the study did not address the issue of longitudinal studies and required a static scan at a later time point, which defeats the benefits of a short early scan. Subsequently, Disselhorst et al. [16] drew attention to the fact that delineation of tumour VOIs may differ significantly depending on time after injection and that this might be an issue for short-scan protocols. This was not a problem in the present study as the VOIs were delineated on CT images and then transferred to the PET dynamic image frames. Peters et al. [17] evaluated a 30-min dynamic [^18^F]NaF PET protocol for assessing bone metabolic activity after spinal fusion surgery by comparing VOIs at the control and operated sites. However, they did not compare their K_i_ results from 30 min scan against the standard 60 min scan due to the infeasibility of long scan times in patients with back pain.

Limitations of this study include the fact that we only studied the short-scan method for measurements in VOIs at the hip, which required previously calculated population average values of parameters from the full 60-min dynamic scan as listed in Table 1. The lumbar spine is also an important site for [^18^F]NaF PET imaging and has the advantage that bone tracer uptake at this site is typically three times greater than at the hip [18], and hence the 60-min Hawkins model rate constants might be more reproducible. Additionally, in the present study, we were unable to compare measurements of the Hawkins model parameter K_1_ between short-scan and conventional 60-min scans. Unlike measurements of bone metabolic flux using 60-min dynamic scans, for which the terminal exponential accounts for 80% of the area under the plasma curve [9], bone blood flow measurements are best performed using individual measurements of the arterial input function over the peak of the bolus injection. Limitations of the short dynamic scan method described here include the fact that additional static scans are required if bone metabolic flux is measured at additional bed positions using the static scan method [6]. However, the overall PET scanning time would still be shorter compared with performing a 60-min dynamic scan.

## 5. Conclusions

We have proposed a dynamic scan method with scan times as short as 12 min for performing [^18^F]NaF PET measurements of bone metabolic flux at the hip that uses fixedrate-constants in the Hawkins model to achieve equivalent or superior statistical power to the conventional 60-min dynamic scan and offers more efficient use of scan time in high demand for routine clinical services.

## Figures and Tables

**Table 1:** Population values of the Hawkins model rate constants in Figure 1A used for the short dynamic scan analyses. The value of K_1_ was set using Equation (1).

Hawkins Model Parameter	Population Values
k_2_	0.194 min^−1^
k_3_	0.187 min^−1^
k_4_	0.014 min^−1^
F_bv_	0.002

## Data Availability

Not applicable.
